# Molecular Characterization and Antimicrobial Susceptibilities of *Nocardia* Species Isolated from the Soil; A Comparison with Species Isolated from Humans

**DOI:** 10.3390/microorganisms8060900

**Published:** 2020-06-15

**Authors:** Gema Carrasco, Sara Monzón, María San Segundo, Enrique García, Noelia Garrido, María J. Medina-Pascual, Pilar Villalón, Ana Ramírez, Pilar Jiménez, Isabel Cuesta, Sylvia Valdezate

**Affiliations:** 1 Reference and Research Laboratory for Taxonomy, National Centre of Microbiology, Instituto de Salud Carlos III, Majadahonda, 28220 Madrid, Spain; gcarrasco@isciii.es (G.C.); sansegundojimenezmaria@gmail.com (M.S.S.); ivorycat3@hotmail.com (N.G.); mjmedina@isciii.es (M.J.M.-P.); pvillalon@isciii.es (P.V.); 2 Bionformatics Unit, Applied Services, Training and Research, Instituto de Salud Carlos III, Majadahonda, 28220 Madrid, Spain; smonzon@isciii.es (S.M.); isabel.cuesta@isciii.es (I.C.); 3 Soil Microbiology Laboratory, Microbiology and Parasitology Department, Pharmacy and Bioanalys Faculty, Los Andes University, Mérida 5101, Venezuela; egarcia@ula.ve (E.G.); ramirezana@ula.ve (A.R.); 4 Genomics Laboratory, Applied Services, Training and Research, Instituto de Salud Carlos III, Majadahonda, 28220 Madrid, Spain; mpsancho@isciii.es

**Keywords:** *Nocardia* spp., *Nocardia cyriacigeorgica*, soil, nocardiosis, *gyrB*, MLSA, WGS, antimicrobial resistance, potential new species/subspecies *N. venezuelensis*

## Abstract

*Nocardia* species, one of the most predominant Actinobacteria of the soil microbiota, cause infection in humans following traumatic inoculation or inhalation. The identification, typing, phylogenetic relationship and antimicrobial susceptibilities of 38 soil *Nocardia* strains from Lara State, Venezuela, were studied by 16S rRNA and *gyrB* (subunit B of topoisomerase II) genes, multilocus sequence analysis (MLSA), whole-genome sequencing (WGS), and microdilution. The results were compared with those for human strains. Just seven *Nocardia* species with one or two strains each, except for *Nocardia cyriacigeorgica* with 29, were identified. MLSA confirmed the species assignments made by 16S rRNA and *gyrB* analyses (89.5% and 71.0% respectively), and grouped each soil strain with its corresponding reference and clinical strains, except for 19 *N. cyriacigeorgica* strains found at five locations which grouped into a soil-only cluster. The soil strains of *N. cyriacigeorgica* showed fewer *gyr*B haplotypes than the examined human strains (13 vs. 17) but did show a larger number of *gyrB* SNPs (212 vs. 77). Their susceptibilities to antimicrobials were similar except for beta-lactams, fluoroquinolones, minocycline, and clarithromycin, with the soil strains more susceptible to the first three (*p* ≤ 0.05). WGS was performed on four strains belonging to the soil-only cluster and on two outside it, and the results compared with public *N. cyriacigeorgica* genomes. The average nucleotide/amino acid identity, in silico genome-to-genome hybridization similarity, and the difference in the genomic GC content, suggest that some strains of the soil-only cluster may belong to a novel subspecies or even a new species (proposed name *Nocardia venezuelensis*).

## Importance

This study highlights hitherto unreported identification, typing, phylogenetic relationships, and antimicrobial susceptibility among soil *Nocardia* strains, with special reference to *N. cyriacigeorgica,* the main species detected and one of the most common causes of human nocardiosis. The work also compares soil strains carrying seven *Nocardia* species with clinical strains isolated from humans, detecting only genetic and antimicrobial susceptibilities differences in *N. cyriacigeorgica.* Our findings suggest that some of the soil *N. cyriacigeorgica* strains detected may, in fact, belong to a new subspecies or even a new species (proposed name *Nocardia venezuelensis*).

## 1. Introduction

*Nocardia* spp. species are found everywhere from sludge and soil to contaminated soil water, deep-sea sediments [1], and desert habitats [2]. Some even infect plants and animals [3,4,5]. They are among the most predominant Actinobacteria of the soil microbiota, including that of the extreme biosphere [6]. The members of *Nocardia* spp. are producers of diverse natural bioactive metabolites [7], such as antimicrobials, enzyme inhibitors, immunomodifiers, and plant growth-promoting substances, etc. [8,9], a result of the physiological and biochemical pressures imposed by the environmental conditions under which they live [10]. Their activity in the degradation of polycyclic aromatic hydrocarbon [11,12] focused on them as potential xenobiotic bioremediators.

Although they cause a number of severe invasive diseases [3,13], the members of *Nocardia* spp. are mainly opportunistic pathogens in humans, usually affecting the lungs, central nervous system, and skin [14,15]. The burden of human nocardiosis differs between geographical locations.

In previous work, *Nocardia* strains were isolated from soil collected at different sites in Lara State (Venezuela) [16], where the prevalence of human mycetoma (a severe cutaneous infection) is high. The present work examines the identity of these strains via 16S rRNA and *gyrB* genes analysis, together with multi-locus sequence analysis (MLSA), whole-genome sequencing (WGS), and susceptibilities. With a special focus on the most prevalent soil species detected, differences and similarities with clinical strains were explored.

## 2. Materials and Methods

### 2.1. Molecular Identification of Species

**Figure 1:**
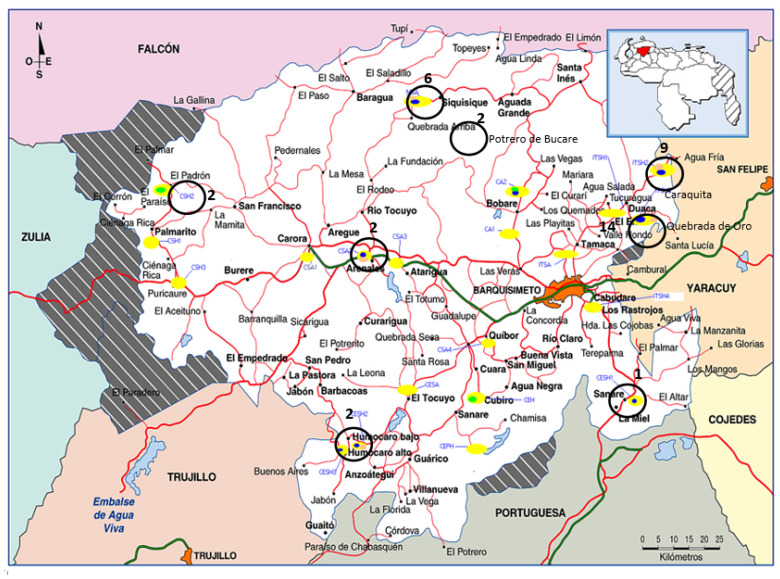
Geographic distribution of soil *Nocardia* strains collected in Lara State (Venezuela). Numbers indicate the number of strains isolated per site.

In the present work, 38 phenotypically identified strains were submitted to the National Centre for Microbiology (CNM, Majadahonda, Madrid, Spain) for molecular identification. The strains were isolated from soil samples collected over two periods-08/2002 and 05/2006-from nine sites in six municipalities in Lara State, NW Venezuela (Figure 1). Table 1 shows the climatic characteristics of each site, and the Supplemental File the soil culture and the phenotypic identification previously described [16]. After growth on Columbia agar supplemented with 5% (*v/v*) sheep′s blood and buffered charcoal–yeast extract agar (BCYE) for 48–72 h at 37 °C under aerobic conditions, their chromosomal DNA was extracted by the boiling method. The 16S rRNA and *gyrB* genes were then amplified and sequenced as previously described [17], and species identified by comparing them against type strain sequences [18,19] using the BLAST algorithm v.2.2.10 (http://www.ncbi.nlm.nih.gov/BLAST). Similarity values of ≥99.6% for 16S rRNA [20], and ≥93.5% for *gyr*B, were deemed to indicate the same species [19]. Sequences were assembled using SEQ-Man software (DNASTAR, Inc., Madison, WI) and, using BioEdit [21], adjusted for phylogenetic analysis to coincide with the length of the shortest sequence for each reference strain (16S rRNA 1215 bp; *gyr*B 726 bp). They were then aligned using the ClustalW algorithm [22], and 16S rRNA and the *gyrB* phylogenetic trees constructed using MEGA 6 software [23] following the neighbor-joining (NJ) and maximum likelihood (ML) methods [24] with 1000 bootstrap replications.

### 2.2. Multilocus Sequence Analysis (MLSA)

All 38 soil strains were then subjected to MLSA [25] alongside a further five Venezuelan strains (three of *N. cyriacigeorgica* and two of *N. farcinica*), eight Spanish clinical *Nocardia* strains, and type strain sequences retrieved from GenBank. MLSA was performed using trimmed sequences of concatenated *gyrB*-16S rRNA-*secA1*-*hsp65* (1790-bp). A NJ phylogenetic tree was then constructed using MEGA 6 software [23]. It should be noted that *N. elegans* lacks a reference type strain for all the genes here examined; the clinical *N. elegans* 20130578 strain was used as an alternative, and it is, therefore this strain that appears in the phylogenetic tree.

### 2.3. Genetic Similarities Among Soil and Clinical Nocardia cyriacigeorgica Strains

Given the strong predominance of *Nocardia cyriacigeorgica* strains in the sampled soils, 30 previously characterized Spanish clinical strains belonging to this species [17] were compared with them in terms of their 16S rRNA, *gyrB*, and GyrB (DNA gyrase subunit B) sequences. Hunter-Gaston discrimination indices (HGDI) [26], single nucleotide polymorphisms (SNPs), and haplotype numbers were examined using DnaSP software [22]. The *N. cyriacigeorgica* 16S rRNA, *gyrB,* and GyrB sequences of the type strain DSM 44484^T^ (GenBank accession number AF430027, GQ496121, and ACV89678, respectively) were used to determine SNP numbers. In addition, the population structures of the soil and clinical groups were examined via a *gyrB* NJ phylogenetic tree, with the inclusion of a further 3 Venezuelan clinical strains and the genome reference strain GUH-2 (GenBank accession number FO082843).

### 2.4. Antimicrobial Susceptibilities

The antimicrobial susceptibilities of all the strains were determined according to CLSI M24-A2 guidelines, using the corresponding control strains [20] and employing the microdilution method with RAPMYCO panels (ThermoFisher, Inc., Cleveland, OH, USA). These panels contain amikacin (AMI), amoxicillin/clavulanic acid (AUG2), cefepime (FEP), cefoxitin (FOX), ceftriaxone (AXO), ciprofloxacin (CIP), clarithromycin (CLA), doxycycline (DOX), imipenem (IMI), linezolid (LZD), minocycline (MIN), moxifloxacin (MXF), tigecycline (TGC), tobramycin (TOB), and co-trimoxazole (trimethoprim/sulfamethoxazole, SXT). Minimum inhibitory concentrations (MIC) were determined following Clinical Laboratory Standard Institute interpretative criteria [27]; intermediate values were categorized as resistant. Susceptibility to trimethoprim/sulfamethoxazole and linezolid was tested using the E-test (bioMérieux, Marcy-l’Étoile, France). Susceptibility rates across strains belonging to the main species from the soil and human sources were compared using the χ^2^ test or two-tailed Fisher’s exact test as required. Significance was set at *p* ≤ 0.05. All calculations were performed using STATA v.13.1 software (StataCorp, College Station, TX, USA).

### 2.5. Bioinformatic Analysis

Six representative soil *N. cyriacigeorgica* strains, thought to be distinct according to their *gyrB* analysis results, were sequenced. Genomic DNA was extracted from single subcultured colonies using the QIAamp DNA Mini Kit (Qiagen, Hilden, Germany). Paired-end libraries were prepared using the Nextera-XT DNA Library Preparation Kit (Illumina 1.9, San Diego, CA, USA) and sequencing performed using the Illumina NextSeq 500 platform (mean sequencing depth ∼90 × per sample). Read quality control was undertaken using FastQC v. 0.11.8 software. Trimmomatic v. 0.33 software [28] was used to remove adapter contamination and to trim low-quality regions (phred >2 0 in a 4 nt window, minimum length 70 bp). Kmerfinder v. 3.0 software [29] was then used for species confirmation and to detect contamination. Assembly was performed using Spades v. 3.8.0 software [30]; Prokka v. 1.12 software [31] was used for genome annotation. Quast v. 4.1 software [32] was used for assembly quality control.

Species assignations were confirmed by comparing the average nucleotide identity (ANI) (https://www.ezbiocloud.net/tools/ani) [33], average amino acid identity (AAI) (http://enve-omics.ce.gatech.edu/aai) [34], and in silico genome-to-genome distance similarity (GGDH; DDH-estimate) (https://ggdc.dsmz.de/ggdc.php#) [35] results against the *N. cyriacigeorgica* GUH-2 (NC_016887.1) reference genome, the genome of the type strain DSM 44484^T^ (NZ_VBUR00000000.1), and other genomes [36]. The AAI-profiler (http://ekhidna2.biocenter.helsinki.fi/AAI) [37], TrueBac^TM^ ID^BETA^ (https://www.truebacid.com/genome/) [38], and the Type Strain Genome Server (TYGS) (https://tygs.dsmz.de) [39] web servers were also used to resolve taxonomic identities.

High-quality assemblies of the same six soil *Nocardia* strains, the type strain DSM 44484^T^, the reference genome of *N. cyriacigeorgica* GUH-2, and five genome assemblies for *N. cyriacigeorgica* (available in NCBI at the time of publication) were subjected to core-genome gene-by-gene typing (cgMLST) using chewBBACA v. 2.0.17.2 software (open-source in https://github.com/B-UMMI/chewBBACA) [40]. Those loci corresponding to potentially complete coding sequences (CDS) that were unique, but present in 95% of the strains, were used in subsequent phylogenetic analysis, using GrapeTree v .2.0 software to visualize the results. A phylogenetic analysis was also performed using bcgTree v .1.1.0 software (available at https://github.com/iimog/bcgTree) [41]; this searches for 107 conserved proteins among the examined bacteria and creates a concatenated gene matrix for a maximum likelihood phylogeny analysis with 100 bootstrap replications (performed using RAxML v .8.2.9 software) [42].

Antimicrobial resistance genes were searched using different tools as (date last accessed, May 2020): ResFinder (identification threshold of 90%) [43], Antibiotic-Resistant Target Seeker (ARTS) [44], the Comprehensive Antibiotic Resistance Database (CARD, with strict criteria in RGI) [45] and by KOALA for KEGG Orthology [46]. Additionally, the SRST2 program [47], was used to detect resistance genes and alleles with the ARGannot database [48]. Phages were identified using PHASTER software (PHAge Search Tool Enhanced Release) (https://phaster.ca/) [49].

### 2.6. Accession Number(s)

This Whole Genome Shotgun project has been deposited at DDBJ/ENA/GenBank under the accession numbers JAAGVC000000000, JAAGVB000000000, JAAGVA000000000, JAAGUZ000000000, JAAGUY000000000 and JAAGUX000000000. https://www.ncbi.nlm.nih.gov/genome/.

## 3. Results

### 3.1. Distribution of Nocardia Species in the Soil

The number of *Nocardia* strains recovered from the soil samples ranged from 1–14 (mean = 6 strains per sample). The Quebrada de Oro (14 strains) and Caraquita (9 strains) sites returned the highest number of soil strains. 16S rRNA sequencing [50] identified the species of all 38 strains with the following distribution: *N. cyriacigeorgica* 29 strains, *N. abscessus* 2, *N. rhamnosiphila* 2, *N. vermiculata* 2, *N. asteroides* 1, *N. elegans* 1, and *N. mexicana* 1 strain. Three different species were found in Quebrada de Oro and Siquisique, in the Crespo and Urdancia municipalities, respectively. The most common species, *N. cyriacigeorgica*, was present at all sites except for El Padrón (in the Torres municipality) (Table 1). Species assignment via *gyrB* analysis [19] agreed with the 16S rDNA-based identifications for 27 strains (71.05%). Table 1 highlights those for which the results were discrepant.

### 3.2. Phylogenetic Analysis by MLSA

**Figure 2:**
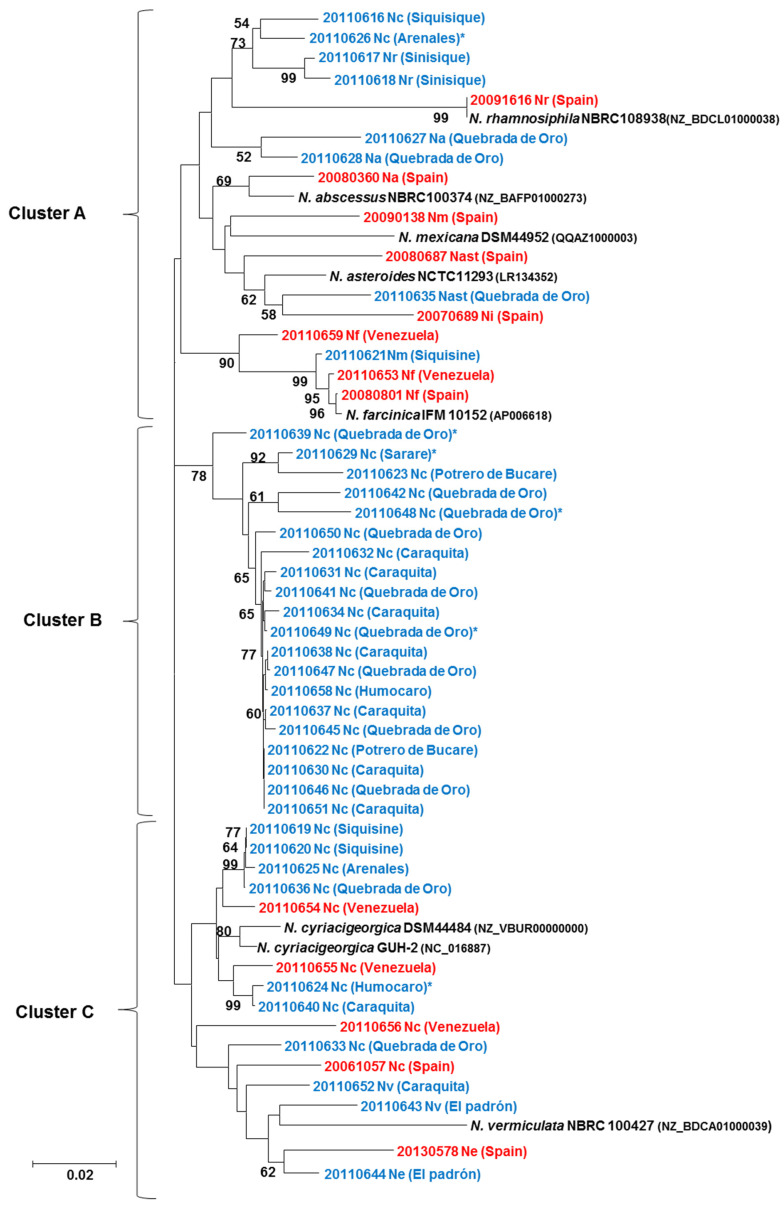
Phylogenetic tree based on the MLSA neighbor-joining (NJ) analysis (*gyrB*-16S rRNA-*secA-hsp65* genes) of the 38 *N. cyriacigeorgica* soil strains (in blue), six Venezuelan and nine Spanish clinical strains (in red), plus the type strains (in black). The asterisk indicates the strains selected for WGS. Na stands for *N. abscessus*, Nast for *N. asteroides*, Nc for *N. cyriacigeorgica*, Ne for *N. elegans*, Nf for *N. farcinica*, Ni for *N. ignorata*, Nm for *N. mexicana*, Nr for *N. rhamnosiphila*, and Nv for *N. vermiculata*. The reliability of the topologies was assessed by the bootstrap method (1000 replications).

MLSA assigned all the soil strains but four to the same species as determined by 16S rRNA analysis (Table 1). The percentage similarity of each MLSA sequence with respect to the MLSA sequence of the respective type strain was: 94.0–98.3% in *N. cyriacigeorgica*, 93.2–94.5% in *N. abscessus*, 93.0–93.5% in *N. rhamnosiphila*, 96.3% in *N. asteroides*, and 93.5% in *N. mexicana*. In addition, MLSA confirmed the *gyrB*-based identification of 27 strains. The MLSA phylogenetic tree showed the 38 soil strains to group into three clusters for NJ (Figure 2), and more for ML topologies (Supplementary Figure S1). Most gathered into cluster A, *N. elegans*, *N. nova* and *N. vermiculata* grouped into cluster C, and *N. cyriacigeorgica* strains were found in all three clusters. The clinical strains fell closer to the type strain of each species than did the soil strains. Twenty of the 29 soil *N. cyriacigeorgica* strains fell into cluster B: the remainder were distributed across clusters C (*n* = 7) and A (*n* = 2).

### 3.3. Antimicrobial Susceptibilities

Table 1 shows the antimicrobial-resistance phenotypes for each soil strain. These soil strains showed a phenotype that fitted the drug pattern type [51], except for the *N. asteroides* soil strain which was susceptible to aminoglycosides and clarithromycin. The *N. mexicana* strain showed a wider resistance spectrum. The soil strains of *N. cyriacigeorgica* showed variable resistance to amoxicillin-clavulanate, clarithromycin, and ciprofloxacin. Table 2 shows the corresponding MIC50, MIC90, MIC range, and resistance rates.

### 3.4. Comparison of Soil and Human N. cyriacigeorgica Strains

Table S1 and Table 2 compare the 16S rRNA, *gyrB,* and GyrB sequences and antimicrobial susceptibilities of the soil *N. cyriacigeorgica* strains to those reported for previously studied Spanish human strains [17]. The soil strains were represented by three 16S haplotypes while the human strains were represented by just one, and by 13 *gyrB* haplotypes rather than 17 for the human strains. The high HGDI of the clinical *N. cyriacigeorgica* strains showed them to be more diverse than the soil strains (0.94 vs. 0.761). However, compared to the type strain DSMZ 44484^T^, the soil strains had higher SNP numbers, and wider SNP ranges per strain, than did the clinical strains (212 vs. 77 and 1–109 vs. 0–38).

**Figure 3:**
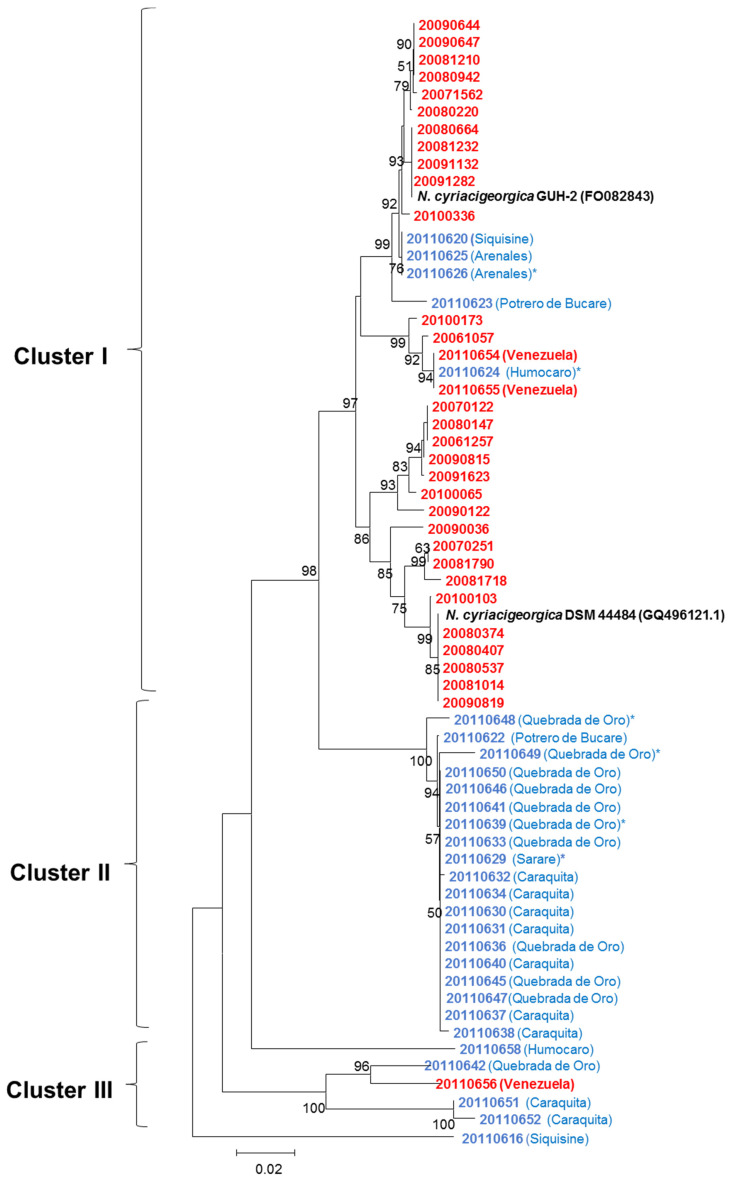
Phylogenetic relationships of the 29 Venezuelan *N. cyriacigeorgica* soil strains (in blue), three Venezuelan and 30 Spanish *N. cyriacigeorgica* clinical strains (in red), as revealed by their *gyrB* genes. The reliability of the NJ topologies was assessed by the bootstrap method (1000 replications). The asterisk indicates the strains selected for WGS.

The *gyrB*-based phylogenetic relationships among the soil and human *N. cyriacigeorgica* strains, the three Venezuelan clinical strains, the type strain DSM 44484^T^, and the genome reference strain GUH-2 are shown in Figure 3 and Supplementary Figure S2. The main cluster (cluster I) includes the 30 Spanish clinical strains, five soil strains, the three Venezuelan clinical strains, plus the two reference strains. Two subclusters with 20 and 17 strains were also seen, with *N. cyriacigeorgica* GUH-2 in one and DSM 44484^T^ in the other. Nineteen of the 29 *N. cyriacigeorgica* soil strains gathered into a soil-only cluster (cluster II), i.e., it contained no human source strains. This cluster showed similarity values ranging from 91.2–92.3% with respect to the type strain. Finally, three soil strains and one clinical strain grouped into a minor cluster with two independent branches (one strain each one).

The soil strains showed low resistance (0–5%) to ceftriaxone, cefepime, imipenem, amikacin, tobramycin, co-trimoxazole, and linezolid, intermediate resistance to minocycline (24%), ciprofloxacin (28%), and amoxicillin-clavulanic acid (48%), and strong resistance to clarithromycin (96.5%) (Table 2). Their susceptibilities to aminoglycosides, doxycycline, tigecycline, co-trimoxazole, and linezolid were similar to those shown by the human strains. However, differences (*p* ≤ 0.05) were seen between the soil and clinical strains for all studied beta-lactams, fluoroquinolones, clarithromycin, and minocycline. Overall, the human *N. cyriacigeorgica* strains were more resistant (except for clarithromycin) than the soil strains. With respect to tigecycline (for which there are no available breakpoints for *Nocardia*), only one soil and three human strains returned MIC values of ≥4 mg/L.

### 3.5. Whole-Genome Sequencing of the soil N. cyriacigeorgica Strains

Whole-genome sequences of six soil *N. cyriacigeorgica* strains were obtained: two belonging to the major *gyrB* cluster (cluster I) and four to cluster II (the soil-only cluster). Their ANI and AAI and in silico GGDH (DDH-estimate) values, genomic G + C percentages, and other characteristics were used to determine their species. The same was performed for other *N. cyriacigeorgica* strains for which genomes were available (Table 3). Although all the strains showed 16S rRNA identities of ≥99.6% with respect to the type strain and the genome of the reference strain [20], the ANI-AAI values for the GUH-2 strain were <95% [52] (except for the ANI of strain 20110626). Strain 20110626, together with 20110624 (both in cluster I), showed higher ANI-AAIs (> 89.84% and > 91.12% respectively) than the other four studied genomes. Determining the DDH-estimate and G + C content via the GGDH server (https://ggdc.dsmz.de/ggdc.php#) [35] showed the four selected strains (20110629, 20110639, 20110648, and 20110649) of the soil-only cluster (cluster II) did not meet the conditions of ≥70% DDH-estimate plus a difference of <1% G + C with respect to strains GUH-2 and DSM 44484^T^; they were therefore interpreted as being ′distinct species′. In addition, lower *gyrB* identity (≤ 93.5%) and G + G content (all 67.2%) values were seen for all the strains of the soil-only cluster than for the strains of cluster I (≥95.3% and ≥68.3% respectively). In contrast, for two strains of cluster I, and for those with available genomes (strains 3012STDY6756504, EML 446, EML 1456, MDA3349, MDA3732) [36], one or more criteria were met, rendering their interpretation as either “distinct or belonging to the same species”, i.e., they could not be clearly identified.

**Figure 4:**
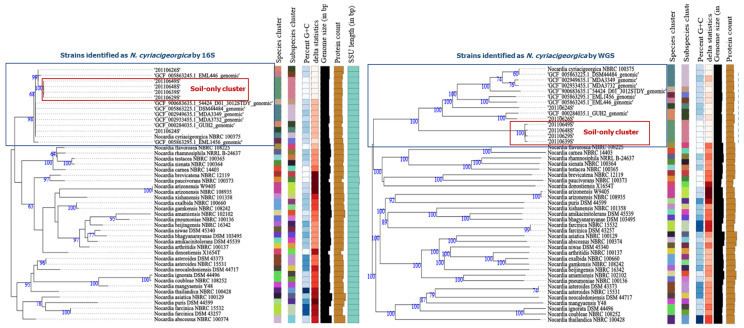
16S rRNA gene sequence-based and whole-genome sequence-based phylogenetic trees constructed using FastME v.2.1.6.1 software (which calculates Genome BLAST Distance Phylogeny (GBDP) distances; the branch lengths are scaled in terms of GBDP distance formula). The numbers above the branches are GBDP pseudo-bootstrap support values (all are >60% from 100 replications), with average branch support of 91.8% and 58.8% for the 16S rRNA gene and for the genome respectively. The trees were rooted at the midpoint. The results were provided by the Type Strain Genome Server (TYGS), a free bioinformatics platform available at https://tygs.dsmz.de (The whole genome-based taxonomic analysis was performed on 8th January 2020) [39].

To check these interpretations, analyses were run using the TrueBac^TM^ ID^BETA^, AAI-profiler, and TSGY [37,38,39] web servers (Supplementary Table S2). Using the TrueBac^TM^ server, the ANI values for three of the four sequenced genomes from the soil-only cluster with respect to the GUH-2 reference genome was 87.7% (0.877). With the AAI-profiler, the AAIs of the four selected strains from the soil-only cluster, and that of the EML 1456 strain, were ~75%; the remainder were over ≥80%. When the TYGS server (https://tygs.dsmz.de) [39] was used to determine AAI with respect to GUH-2 and DSM 44484^T^, the strains of the soil-only cluster returned > 1% difference in the G + C content; no such result was returned for any other strain. When these four selected strains were compared among themselves, the *gyrB*, ANI, AAI, DDH, and G + C ranges were 97.8–98.8%, 99.65–99.73%, 99.56–99.73%, 97.70–98.80%, and 0.01–0.33, respectively. Also using the TYGS, 16S rRNA gene sequence-based and whole-genome sequence-based trees were constructed with the above-mentioned genome sequence data and those of *Nocardia* type strains of other species. The soil-only cluster appeared separated from the other *N. cyriacigeorgica* strains in the whole-genome sequence-based tree (Figure 4).

**Figure 5:**
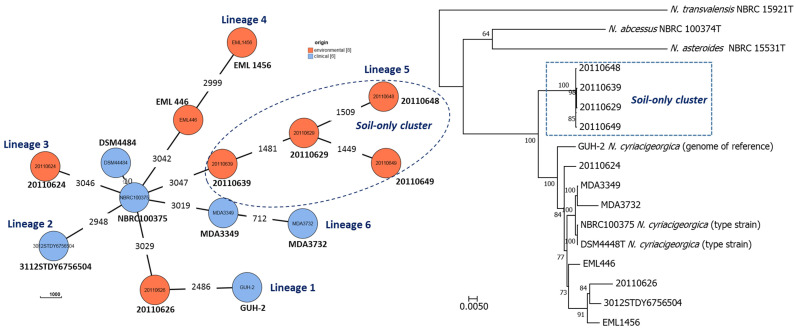
Left: Phylogenetic tree constructed by MAFFT alignment and neighbor-joining with the Clustal W2 algorithm, based on the cgMLST associations among the *N. cyriacigeorgica* genomes. The tree was built using chewBBACA software and based on 3048 loci. [40]. The DSM 44484T and NBRC 100375 genomes correspond to the *N. cyriacigeorgica* type strain IMMIB D-1627; GUH-2 is the reference genome. Branches indicate the number of different alleles. Right: maximum likelihood phylogenetic tree produced with a concatenated gene matrix with 107 conserved proteins using RAxML v. 8.2.9 and bcgTree software v.1.1.0 software (100 bootstrap replications) [41]. The *N. cyriacigeorgica* soil strains are colored blue and the Nocardia clinical strains red. The percentage of bootstrap replicate trees (1000 replications) in which the associated taxa clustered together are shown next to the branches. Bar: 0.02 changes per nucleotide position.

Using the chewBBACA platform, a novel cgMLST typing method based on 3048 loci was performed independent of any defined comparator strain [40]. The *N. cyriacigeorgica* type strain designated IMMIB D-1627 has several culture collection denominations, including DSM 44484 and NBRC 100375 (although their respective genomes differ in 10 alleles). In the cgMLST dendrogram, the genome of the type strain NBRC 100375 has a central node from which other genomes emerge. Indeed, moving in a clockwise fashion, six distinct lineages can be seen (Figure 5), with the genome of the reference GUH-2 appearing as lineage 1 (with 3034 different alleles). The genomes of the soil strains appeared as lineages 1, 3, and 5, with lineage 5 belonging to the soil-only cluster. The strains of this latter cluster differ in 3047 alleles with respect to the central node of NBRC 100375, and among themselves by a mean 1594 alleles. Using the 107 essential single-copy genes extracted by BCGtree analysis [41], the four selected strains of the soil-only cluster grouped into one of two clusters with a high bootstrap value (Figure 5).

By the use of different platforms [43,44,47,48], the *ast-1* beta-lactamase gene (class A beta-lactamase) was detected in strain with decreased amoxicillin-clavulanate acid susceptibility, CNM20110626, which showed a 98.06% of identity (new allele with 6 amino acids changes) respective to its counterpart in *N. cyriacigeorgica* GUH-2 strain. As well as van*RS*, the two-component system response regulator of the glycopeptide resistance gene cluster, in the NCBR 100375, 3012STDY6756504, MDA3349, EML446, MDA3732 and EML1456 strains. With KOALA for KEGG Orthology [46], more putative antimicrobial genes were identified in both groups of strains as some aminoglycoside resistance genes (*str*B, streptomycin 6-kinase; *aad*A, streptomycin 3”-adenylyltransferase; and *aph*3-II, aminoglycoside 3′-phosphotransferase II), macrolide resistance genes (*erm*C/A, 23S rRNA methyltransferases; *car*A, transport system ATP-binding/permease protein; *ere*A_B, erythromycin esterase; *vat*, virginiamycin A acetyltransferase; *vgb,* virginiamycin B lyase), chloramphenicol resistance protein (*cml*R, MFS transporter), vancomycin resistance (vanY, zinc D-Ala-D-Ala carboxypeptidase), and multidrug resistance efflux pumps genes of MexJK-OprM, MexPQ-OpmE, and QacA). The chloramphenicol 3-O phosphotransferase *cpt*, was detected in GUH-2 and 3012STDY6756504 strains. To note, the *aad*A gene was detected in strains of soil-only cluster strains together with *str*B, but not with *aph*3-II. The remaining strains only have *aph*3-II with *str*B, except CNM20110624 and 3012STDY6756504 strains with *aad*A/*aph3*-II/ *str*B genes.

Regarding the quinolone resistance, the topoisomerase subunits GyrA/B of the soil *N. cyriacigeorgica* strains showed both two major alleles (19 and 11 changes, respectively): GyrA1 and GyrB1, in CNM20110624-626 (with 6 and 4 differences outside of the quinolone-determining-region between both strains); and GyrA2 and GyrB2, without changes in the four strains of the soil-only cluster (Supplementary Figure S3). Lastly, no intact or questionable phages were detected in the soil strains.

## 4. Discussion

Like other actinomycetes, *Nocardia* spp. contribute to soil health, playing major roles in the cycling of organic matter, inhibiting the growth of plant pathogens, and decomposing complex mixtures of dead plants and animals [1,53]. As well as maintaining the biotic equilibrium of the soil, these bacteria are involved in a wide array of opportunistic infections in both immunocompromised and immunocompetent persons [13]. Mycetoma and pulmonary nocardiosis, respectively caused by traumatic inoculation and inhalation, are the most common [14,15]. The increase in the size of the immunocompromised and immunosenescent populations has led to an increase in the number of cases of nocardiosis recorded. The annual incidence rate in Canada has now reached 0.87/100,000 inhabitants [15]; in Western Europe, the hospitalization rate due to nocardiosis has reached 0.04/100,000 inhabitants [54].

Climate, vegetation type, and soil pH probably affect the frequency and diversity of soil aerobic Actinobacteria [55]. Those that cause human infections in any given area are typically those found in the local soil [56]. Thus, different *Nocardia* species appear as major aetiological agents in different countries. For instance, *N. farcinica* causes infections in Canada [15], France [57], and Japan [58], but not in Spain, where the incidence *N. cyriacigeorgica* is double that of *N. farcinica* [59]. *Nocardia* spp. in the environment, thus posing some risk to human health [14], a fact reflected in the greater incidence of mycetoma in farmers and other people from rural areas of Lara State [16].

*Nocardia* genus contains about 200 species (https://lpsn.dsmz.de/), however, in the present work, only seven species were identified, with *N. cyriacigeorgica* the most common (71.8%). Surprisingly, *Nocardia brasiliensis*, the main causal agent of mycetoma in Lara State, was not isolated in the previous work [16]. In south-eastern Spain, *N. cyriacigeorgica* (previously identified as the *N. asteroides* complex) [60] has been detected in soil samples [61], and it is responsible for the majority of human nocardiosis (25%) [59]. However, *N. brasiliensis*, which is responsible for more than half of soft tissue/bone infections [59], was not detected in the above study [61]. This might be explained in that actinomycetes are 3–5.6 times more abundant in air samples above ground than in the soil [62].

With respect to the present molecular targets, MLSA (*gyrB*-16S rRNA-*secA1-hsp65*) was the arbiter of *Nocardia* species identification [25], confirming the 16S rRNA- and *gyrB*-based assignment results for 89.47% and 71.05% of the strains, respectively. Nearly 70% of the soil *N. cyriacigeorgica* strains isolated from five of the nine sampling sites gathered into MLSA cluster B or *gyrB* cluster II (the soil-only cluster). Both clustering methods are valuable in species/subspecies identification [63], although the *gyrB* method, with just one studied gene, is more simple.

*gyrB* gene sequencing showed the soil-derived *N. cyriacigeorgica* strains to be less diverse (lower HGDI) than the human-isolated strains, although the number and range of SNPs per strain were significantly greater. The difference in SNPs found between the DSM 44484^T^ strain and the soil strains might suggest the presence of some atypical *N. cyriacigeorgica* strains. In addition, the soil strains were more susceptible to beta-lactams, fluoroquinolones, and minocycline than were the human strains, and more resistant to clarithromycin. Regarding fluoroquinolones, susceptibility differences could be related to variations in the amino acid composition of GyrA/GyrB. These differences might be the result of reduced exposure to antimicrobials in Venezuelan soils, or perhaps low intrinsic resistance of this variant.

To check the species assignment of the strains in the soil-only cluster - despite them belonging to the same species according to their 16S rRNA results - some were subjected to WGS along with others from outside this cluster. Several coefficients were required to reach specific thresholds for an assignment to be deemed correct: > 95% ANI/AAI, > 70% DDH-estimate and a <1% difference in G + C content [34,52,64,65]. ANI resolves well between genomes that share 80–100% identity, and AAI does so for species that share <80% ANI and/or when 30% of their gene content is very divergent [34]. In the present work, the results of both AAI and ANI were taken into account, along with the DDH-estimate, and the G + C content since a query genome with an ANI of <95% likely represents a new species [66]. Indeed, with respect to the genome of reference strain GUH-2, average ANI and AAI values of around 87% were returned for the strains of the soil-only cluster, along with a mean DDH-estimate of 31.6% and G + C content differences of around 1.5%. In addition, the *N. cyriacigeorgica* strains of the soil-only cluster showed the greatest identity among themselves, with average ANI values of 99.7% being returned. For two strains from outside of the soil-only cluster, as well as for those for which genomes were available, the ANI and AAI values were around 90% and 92%, satisfying the criterion of a <1% difference in the G + C content, meaning they belong to the same species.

According to the commercial TrueBac^TM^ system, and the AAI-profiler and TSGY systems (both open source) [37,38,39], the genomic evidence might suggest that a new species (*Nocardia venezuelensis* sp. nov) exists among the soil-only cluster strains examined, all of which had low G + C contents. Some of the available genomes studied might also belong to a new species. In cgMLST (performed using chewBBACA software) [40], six lineages appear for 12 *N. cyriacigeorgica* strains around the reference strain DSM 44484^T^/ NRBC 100375. It may be that the DSM 44484^T^ strain provides a better reference genome than the current GUH-2 reference strain. Intraspecies MLSA sub-clusters of *N. cyriacigeorgica* have already been described [63]; thus, whole-genome analyses of *N. cyriacigeorgica* should be performed to determine its lineages, with the description of different species/subspecies as members of a single complex.

In conclusion, no genetic differences, nor differences in antimicrobial susceptibilities, were found between the *Nocardia* strains isolated from the Venezuelan soil samples and the reference or clinical strains–except for the strains of *N. cyriacigeorgica*-. This might indicate that some of the latter belong to a new subspecies of *N. cyriacigeorgica* or even a new species. Should this be confirmed, the name *Nocardia venezuelensis* is proposed.

## Figures and Tables

**Table 1:** Characteristics of the *Nocardia* spp. soil strains isolated in Lara State, Venezuela.

Strain No.	Species (16S rDNA)	Percentage Identity with Respect to DMS 44484^T^ 16S rDNA	Percentage Identity with Respect to DMS 44484^T^ *gyrB*	Drug Resistance Phenotype	Location (Municipality)	Latitude (N)/Longitude(W) and Altitude	Temperature/Rainfall	Sample Time (m, yr)	Weather Type
20110625	*N. cyriacigeorgica*	99.84%	99.4%	CIP CLA	Arenales (Torres)	10° 9′11"	27 °C/400mm	August, 2002	Semi-arid continental
20110626 ^b,c,d^	*N. cyriacigeorgica*	100%	95.9%	xl CIP CLA	Arenales	69° 54´12" 517m			
20110630	*N. cyriacigeorgica*	99.84%	93.1%	Xl CLA	Caraquita(Crespo)	10° 41′ 11" 69° 05′ 11" 685m	25 °C/743mm	April, 2006	Subhumid interior (transitional)
20110631	*N. cyriacigeorgica*	99.84%	93.1%	Xl CLA	Caraquita				
20110632	*N. cyriacigeorgica*	99.84%	93.0%	XL cla	Caraquita				
20110634	*N. cyriacigeorgica*	99.84%	93.1%	XL CLA	Caraquita				
20110637	*N. cyriacigeorgica*	99.84%	93.1%	CLA	Caraquita				
20110638	*N. cyriacigeorgica*	99.84%	92.8%	xl	Caraquita				
20110640	*N. cyriacigeorgica*	98.85%	93.1%	CLA	Caraquita				
20110651 ^a,c^	*N. cyriacigeorgica*	99.84%	86.2%	XL CLA CIP	Caraquita				
20110652 ^a,c^	*N. vermiculata*	-	-	-	Caraquita				
20110643 ^a,c^	*N. vermiculata*	-	-	CIP	El Padrón (Torres)	10° 20′ 44″ 70° 28′ 59′″ 643m	26 °C/921mm	May, 2006	Subhumid continental
20110644 ^a,b,c^	*N. elegans*	-	-	XL TOB CIP	El Padrón				
20110624 ^d^	*N. cyriacigeorgica*	99.92%	95.9%	CLA	Humocaro (Morán)	9^o^ 40´57" 69^o^ 58 12" 964m	24 °C/700mm	August, 2002	Subhumid continental (seasonal)
20110658	*cyriacigeorgica*	99.84%	87.9%	Xl CLA	Humocaro				
20110622	*N. cyriacigeorgica*	99.84%	93.3%	CLA CIP	Potrero de Bucare (Iribarren)	10^o^ 18´51" 69^o^ 27´45" 711m	24 °C/700mm 25 °C/339mm		Semi-arid continental
20110623	*N. cyriacigeorgica*	99.75%	98.2%	CIP				August, 2002	
20110627 ^a,c^	*N. abcessus*	-	-	IMI CIP	Quebrada de Oro (Crespo)	10° 16′ 2" 69° 2′ 22" 1278m	24 °C/1285mm	April, 2006	Subhumid interior (transitional)
20110628	*N. abcessus*	-	-	IMI CIP	Quebrada de Oro				
20110633	*N. cyriacigeorgica*	99.84%	93.1%	CLA	Quebrada de Oro				
20110635 ^a,c^	*N. asteroides*	-	-	-	Quebrada de Oro				
20110636	*N. cyriacigeorgica*	99.92%	93.1%	CIP cla	Quebrada de Oro				
20110639 ^d^	*N. cyriacigeorgica*	99.92%	92.6%	CIP CLA	Quebrada de Oro				
20110641	*N. cyriacigeorgica*	99.84%	93.1%	CLA	Quebrada de Oro				
20110642 ^a,c^	*N. cyriacigeorgica*	99.84%	85.1%	xl	Quebrada de Oro				
20110645	*N. cyriacigeorgica*	99.84%	93.1%	XL CLA min	Quebrada de Oro				
20110646	*N. cyriacigeorgica*	99.84%	93.1%	XL CLA CIP	Quebrada de Oro				
20110647	*N. cyriacigeorgica*	100%	93.1%	CLA	Quebrada de Oro				
20110648 ^d^	*N. cyriacigeorgica*	99.84%	92.8%	CLA	Quebrada de Oro				
20110649 ^d^	*N. cyriacigeorgica*	99.84%	91.2%	CLA	Quebrada de Oro				
20110650	*N. cyriacigeorgica*	99.92%	93.1%	XL, CLA	Quebrada de Oro				
20110629 ^d^	*N. cyriacigeorgica*	99.75%	92.4%	CLA	Sarare (Simón Planas)	9° 47′ 2″ 69° 9′ 40″ 269m	26 °C/1434mm	August, 2002	Subhumid continental (seasonal)
20110616 ^b,c^	*N. cyriacigeorgica*	100%	85.0%	CIP, CLA	Siquisique (Urdancia)	10^o^ 34´24" 69^o^ 42´ 5" 271m	27 °C/358mm	August, 2002	Semi-arid continental
20110617 ^a,c^	*N. rhamnosiphila*	-	-	CLA	Siquisique				
20110618 ^a,c^	*N. rhamnosiphila*	-	-	-	Siquisique				
20110619	*N. cyriacigeorgica*	99.92%	99.4%	XL CLA CIP	Siquisique				
20110620	*N. cyriacigeorgica*	100%	99.4%	XL CLA	Siquisique				
20110621 ^a,b^	*N. mexicana*	-	-	Xl IMI tob CLA min	Siquisique				

The vegetation at all sites was thorny scrub, except for the Caraquita, Quebrada de Oro, and El Padrón site, which was forested. The minimum inhibitory concentrations (MIC) values were categorized following the Clinical Laboratory Standard Institute interpretative criteria (CLSI, 2018). Resistant and intermediate values are coded in capital and lowercase respectively. Antimicrobial acronyms: amoxicillin/clavulanate (XL), tobramycin (TOB), clarithromycin (CLA), minocycline (MIN), ciprofloxacin (CIP), trimethoprim/sulfamethoxazole (SxT). ^a^ Disagreement in identification between the 16S rRNA and *gyrB* techniques; ^b^ disagreement in identification between the 16S rRNA and multilocus sequence analysis (MLSA) techniques; ^c^ disagreement in identification between the *gyrB* and MLSA techniques; ^d^ strains studied by whole-genome sequencing (WGS).

**Table 2:** Antimicrobial susceptibilities of 29 *N. cyriacigeorgica* soil strains and 30 *N. cyriacigeorgica* clinical strains. Comparison of resistance rates.

Antimicrobial Agent	MIC (mg/L) ^1^			Resistance (%) ^2–4^	Sign. Difference
	Range	MIC50	MIC90		(*p* ≤0.05)
**Amoxicillin-clavulanic acid ^4^**					
**Soil**	≤2–32	8	32	14 (48.27%)	yes
**Clinical**	≤2–64	32	32	23 (76.7%)	
**Cefoxitin**					
**Soil**	≤4–128	8	32	12 (41.4%) ^5^	yes
**Clinical**	≤4–≥128	128	≥128	28 (93.4%) ^5^	
**Ceftriaxone**					
**Soil**	≤4	≤4	≤4	0	yes
**Clinical**	≤4–16	≤4	8	5 (16.7%)	
**Cefepime**					
**Soil**	≤1–16	2	8	1 (3.4%)	yes
**Clinical**	≤1–32	16	32	18 (60.0%)	
**Imipenem**					
**Soil**	≤2–4	≤2	≤2	0	yes
**Clinical**	≤2–32	8	32	24 (79.2%)	
**Amikacin**					
**Soil**	≤1–16	≤1	2	1 (3.4%)	no
**Clinical**	≤1–16	≤1	≤1	1 (3.0%)	
**Tobramycin**					
**Soil**	≤1–2	≤1	≤1	0	no
**Clinical**	≤1–16	≤1	≤1	2 (6.6%)	
**Ciprofloxacin**					
**Soil**	≤0.12–≥4	1	≥4	8 (28.5.0%)	yes
**Clinical**	2–≥4	≥4	≥4	29 (96.7%)	
**Moxifloxacin**					
**Soil**	≤0.25–≥4	0.5	4	10 (34.5%)	yes
**Clinical**	1–≥4	4	≥4	29 (96.7%)	
**Clarithromycin**					
**Soil**	1–≥16	8	16	28 (96.5%)	yes
**Clinical**	≤0.06–≥16	≥16	≥16	22 (73.3%)	
**Doxycycline**					
**Soil**	≤0.25–8	2	4	17 (58.6%)	no
**Clinical**	≤0.12–8	2	4	19 (62.7%)	
**Minocycline**					
**Soil**	≤1–8	≤1	2	7 (24.1%)	yes
**Clinical**	≤1–4	2	4	18 (59.4%)	
**Tigecycline**					
**Soil**	0.06–4	0.25	1	-^6^	no
**Clinical**	≤0.25–≥4	0.25	2	-^6^	
**Co-trimoxazole ^4^**					
**Soil**	≤0.25–0.5	0.25	0.5	0	no
**Clinical**	≤0.25–4	0.5	2	1 (3.3%)	
**Linezolid**					
**Soil**	≤1–2	≤1	≤1	0	no
**Clinical**	≤1–4	≤1	2	0	

^1^ Minimum inhibitory concentrations, MIC_50_ and MIC_90_ are the MICs at which 50% and 90% of the strains were inhibited respectively. ^2^ Clinical Laboratory Standard Institute intermediate and resistant criteria (document M24-A2) (values expressed in mg/L): amoxicillin/clavulanate, cefepime and cefoxitin (XL, FEP, FOX, 16, ≥32); ceftriaxone (AXO, 16–32, ≥64); imipenem (IMI, 8, ≥16); amikacin (AMI, -, ≥16); tobramycin (TOB, 8, ≥16); ciprofloxacin and moxifloxacin(CIP and MXF, 2, ≥4); clarithromycin (CLA, 4, ≥ ); doxycycline and minocycline (DOX and MIN, 2–4, ≥8); trimethoprim/sulfamethoxazole (SXT, -, ≥4/76); linezolid (LZD, ≥16); ^3^ Number and percentage of intermediate and resistant strains; ^4^ Concentrations of amoxicillin/clavulanate (ratio 2:1) and trimethoprim/sulfamethoxazole (ratio 1:19) are expressed in terms of amoxicillin and trimethoprim respectively; ^5^ The available breakpoint for cephalosporins was used (≥8 mg/L); ^6^ No available breakpoint.

**Table 3:** Comparison of the whole-genome sequences of the *N. cyriacigeorgica* soil strains and other stated strains, with respect to the reference genome of *N. cyriacigeorgica* GUH-2 (NC_016887.1) and the genome of the type strain *N. cyriacigeorgica* DSM 44484^T^.

Strain (ID/refSeq)	G + C% Length (no. of Contigs; Depth Coverage)	16S rRNA (≥ 99.6%) ^1,2^		*gyrB* (≥ 93.5%) ^1,2^		ANI (≥ 95%) ^1,2,3^		AAI (≥ 95%??) ^1,2,4^		DDH-Estimate (GLM-based) (≥70% DDH-Estimate, Difference in <1% G + C) ^1,2,5^ and Interpretation	
Strain for Comparison		GUH-2	DSM 44484^T^	GUH-2	DSM 44484^T^	GUH-2	DSM 44484^T^	GUH-2	DSM 44484^T^	GUH-2	DSM 44484^T^
GUH-2 NC_016887	68.37% 6,194,645 (1)	--	100	--	94.77	--	*90.14*	--	92.08	--	99.9% (99.8–100%) 0.03 (either distinct or same species)
DSM 44484^T^ NZ_VBUR00000000.1	68.19% 6,311,306 (1, 484x)	100	--	94.77	*--*	*90.14*	--	*92.08*	-	99.9% (99.8–100%) 0.03 (either distinct or same species)	--
**Soil Strains**											
20110624 JAAGVC000000000	68.39% 6,326,508 (113;79x)	100	100	95.32	95.98	*89.84*	*91.12*	*91.12*	*93.11*	*38.0*% (36.3–41.3%) 0.05 (either distinct or same species)	43.6% (41.1–46.1%) 0.10 (either distinct or same species)
20110626 JAAGVB000000000	68.29% 6,578,812 (158;100x)	99.79	99.87	99.45	95.34	97.84	*90.64*	*91.74*	*92.03*	86.0% (83.4–88.3%) 0.1 (either distinct or same species)	*41.2*% (38.7–3.8%) 0.04 (either distinct or same species)
20110629 JAAGVA000000000	66.87% 6,251,294 (71;154x)	99.79	99.87	*93.12*	*92.37*	*86.77*	*86.38*	*88.21*	*88.23*	*31.5*% (29.1–34%) *1.75* (distinct species)	*31.4*% (29–33.9%) *1.61* (distinct species)
20110639 JAAGUZ000000000	66.95% 6,200,016 (178;45x)	99.79	99.87	*93.12*	*92.6*	*86.57*	*85.85*	*88.22*	*88.30*	*31.6*% (29–34.1%), *1.43* (distinct species)	*31.5*% (29.1–34%) *1.29* (distinct species)
20110648 JAAGUY000000000	66.96% 6,274,061 (57;126x)	99.79	99.87	*92.85*	*92.85*	*86.62*	*85.68*	*88.21*	*88.21*	*31.6*% (29.2–34.1%) *1.42* (distinct species)	*31.5*% (29.1–34%) *1.28* (distinct species)
20110649 JAAGUX000000000	66.92% 6,258,095 (135;46x)	99.79	99.87	*92.16*	*91.20*	*86.72*	*85.81*	*88.21*	*88.22*	*31.6*% (29.2–34.1%) *1.46* (distinct species)	*31.5*% (29.1–34%) *1.31* (distinct species)
**Strains with available genome**											
3012STDY6756504 NZ_LR215973.1	68.20% 6,476,621 (1535;100x)	100	100	96.84	97.93	*89.96*	96.26	*92.26*	96.82	39.9% (37.4–42.4%) 0.13 (either distinct or same species)	68.8% (65.8–71.6%) 0.04 (either distinct or same species)
EML 446 NZ_VBUT00000000.1	68.20% 6,520,205 (14;463x)	100	100	97.08	97.03	*90.34*	*91.99*	*92.42*	*93.72*	41.1% (38.6–43.6%) 0.14 (either distinct or same species)	47.0% (44.4–49.6%) 0.03 (either distinct or same species)
EML 1456 NZ_VBUU00000000.1	68.00% 6,830,276 (108;458x)	100	100	96.94	96.95	*90.26*	*92.07*	*92.37*	*93.73*	40.9% (38.4–43.4%) 0.34 (either distinct or same species)	47.2% (44.6–9.8%) 0.17 (either distinct or same species)
MDA3349 NZ_CP026746.1	68.30% 6,462,637 (9; 43x)	100	100	96.55	99.84	*90.21*	97.83	*92.30*	98.00	41.3% (38.8–3.9%) 0.15 (either distinct or same species)	81.2% (78.3–83.8%) 0.09 (either distinct or same species)
MDA3732 NZ_PSZF00000000.1	68.29% 6,592,249 (84;172x)	100	100	94.32	96.97	*90.56*	97.62	*92.46*	97.87	39.9% (37.4–42.4%) 0.13 (either distinct or same species)	80.3% (77.4–83%) 0.03 (either distinct or same species)

^1^ The reference breakpoints for assigning membership to a specific species for 16S rRNA, *gyrB*, average nucleotide identity (ANI), average amino acid identity (AAI), in silico genome-to-genome distance similarity (GGDH; DDH-estimate) and a difference in G + C content, are indicated in brackets in the column headings. ^2^ Values lower than the reference breakpoints, suggestive of a distinct species, are indicated in italics. ^3^ ANI and coverage (range 50.94-63.08) were determined using the EzBioCloud platform (https://www.ezbiocloud.net/tools/ani). ^4^ AAI at the Kostas Laboratory (http://enve-omics.ce.gatech.edu/aai). ^5^ DDH-estimate and difference in genomic G + C content using the DSMZ platform (https://ggdc.dsmz.de/ggdc.php#).

## Supplementary Materials

The following are available online at https://www.mdpi.com/2076-2607/8/6/900/s1, Figure S1. Phylogenetic ML tree based on the MLSA analysis gyrB-16S rRNA-secA-hsp65 genes) of the 38 N. cyriacigeorgica strains from soil (in blue), 5 Nocardia clinical strains from Venezuelan patients and nine Spanish clinical strains representing each species present in soil (in red), plus the type strains (in black). The asterisk indicates the strains selected for WGS. Na stands for N. abscessus, so on Nast for N. mexicana, Nc for N. cyriacigeorgica, Ne for N. elegans, Nf for N. farcinica, Ni for N. ignorata, Nm for N. mexicana, Nn for N. nova, Nr for N. rhamnosiphila, and Nv for N. vermiculata. The reliability of the topologies was assessed by the bootstrap method with 1000 replicates; Figure S2. Phylogenetic relationships of the 29 Venezuelan N. cyriacigeorgica soil strains (in blue), three Venezuelan and 30 Spanish N. cyriacigeorgica clinical strains (in red), as revealed by their gyrB genes. The reliability of the ML topologies was assessed by the bootstrap method (1000 replications). The asterisk indicates the strains selected for WGS; Figure S3. Amino acid sequences of GyrA alignment from N. cyriacigeorgica genome reference strain GUH-2, type strain DSM44484T, CNM20110626, and CNM20110624 with major allele GyrA1, and the strains of the soil-only cluster (CNM20110629, CNM20110639, CNM20110648, and CNM20110649) with major allele GyrA2; Table S1. Comparison of the main typing characteristics (16S rDNA, gyrB, and GyrB) between the N. cyriacigeorgica soil strains from Lara State (Venezuela) and clinical samples from Spanish patients; Table S2. Interpretations of the analysis of the genomes of the soil N. cyriacigeorgica strains and the NCBI-available N. cyriacigeorgica genomes in terms of gyrB, ANI, AAI, in silico genome-to-genome distance similarity (GGDH; DDH-estimate), and differences in G+C content.
