# Supplementary material for: Molecular Characterization and Antimicrobial Susceptibilities of Nocardia Species Isolated from the Soil; A Comparison with Species Isolated from Humans
Source: Microorganisms. 2020 Jun 15;8(6):900. doi: 10.3390/microorganisms8060900 (PMC7355893; doi:10.3390/microorganisms8060900)
Supplement: Supplementary file 1 [file microorganisms-08-00900-s001.pdf]

Table S1. Comparison of the main typing characteristics (16S rDNA, *gyr*B, and GyrB) between the *N. cyriacigeorgica* soil strains from Lara State (Venezuela) and clinical samples from Spanish patients.

**Target**	**16S rRNA (514bp*)**		***gyrB* (726bp*)**		**GyrB (242aa†)**	
**Source (no. of strains)**	***Soil samples* (n=29)**	***Clinical samples* (n=30)**	*Soil samples* (n=29)	*Clinical samples* (n=30)	*Soil samples* (n=29)	*Clinical samples* (n=30)
**Haplotypes number**	3	1	13	17	12	9
**HGDI‡**	0.537	0	0.761	0.940	0.726	0.786
**SNPs number¥ (divergence rate)**	2 (0-1)	0	212 (0.0-16.4)	77 (0.0–5.6)	121 (0.0-1.8)	12 (0.0-0.4)
**SNP range per strain¥ (average, mode)**	0-1 (0.1, 0)	0	1-109 (43, 50)	0-38 (28, 36/38)	2-72 (30, 37)	0-10 (6, 7)

*Analyzed sequence size in numbers of base pairs; †Analyzed sequence size in numbers of amino acids; ‡HGDI correspond to the Hunter and Gaston discrimination index; ¥With respect to *N. cyriacigeorgica* DSM 44484 (GenBank accession number NR_041857 for 16S rRNA, and JN041323 for *gyrB* and GyrB).

Table S2. Interpretations of the analysis of the genomes of the soil *N. cyriacigeorgica* strains and the NCBI-available *N. cyriacigeorgica* genomes in terms of *gyr*B, ANI, AAI, *in silico* genome-to-genome distance similarity (GGDH; DDH-estimate), and differences in G+C content.

**Strain (ID no./refSeq)**	***gyrB* (≥93.5%)[1,2]**		**TrueBac™ ANI[3]**	**AAI profiler[4] (average identity)**	**Type (Strain) Genome Server[5] (≥70% DDH-estimate, difference of <1% G+C)[1,2] and interpretation**	
	**GUH-2**	**DSM 44484^{T}**	**GUH-2**	**GUH-2**	**GUH-2**	**NBRC 100375 (alternative designation DSM 44484^{T})**
GUH-2 (NC_016887)	--	94.77	Not determined[6]	--	--	0.17<br>Potential new species
DSM 44484^{T} (NZ_VBUR00000000)	94.77	--	Not determined[6]	0.93	0.15	0.03<br>Belongs to *N. cyriacigeorgica*
20110624 (JAAGVC000000000)	95.32	95.98	*90.26*<br>*Nocardia* sp. nov. genomic evidence	*0.80*	0.05	65.9 - 73.5, 0.13<br>Potential new species
20110626 (JAAGVB000000000)	99.45	95.34	98.34<br>*Nocardia* sp. nov.	*0.85*	0.11	61.2 - 68.6, 0.06<br>Potential new species
20110629 (JAAGVA000000000)	*93.12*	*92.37*	*87.76*<br>*Nocardia* sp. nov<br>*cyriacigeorgica GUH-2 (FO082843)*	*0.76*	*1.75*	44.8 - 51.7, *1.58*<br>Potential new species
20110639 (JAAGUZ000000000)	*93.12*	*92.6*	*87.78*<br>*Nocardia* sp. nov.	*0.76*	*1.43*	45.2 - 52.1, *1.26*<br>Potential new species
20110648 (JAAGUY000000000)	*92.85*	*92.85*	*87.78*<br>*Nocardia* sp. nov.	*0.75*	*1.42*	45.0 - 51.9, *1.25*<br>Potential new species
20110649 (JAAGUX000000000)	*92.16*	*91.20*	Not determined[6]	*0.75*	*1.46*	44.7 - 51.5, 1.28<br>Potential new species
3012STDY6756504 (NZ_LR215973)	96.84	97.93	Not determined[6]	*0.82*	0.13	80.9 - 87.8, 0.04<br>Potential new species
EML 446 (NZ_VBUT00000000)	97.08	97.03	Not determined[6]	*0.80*	0.14	66.0 - 73.5, 0.03<br>Potential new species
EML 1456 (NZ_VBUU00000000)	96.94	96.95	Not determined[6]	*0.75*	0.34	61.1 - 68.5, 0.17<br>Potential new species
MDA3349 (NZ_CP026746)	96.55	99.84	Not determined[6]	*0.82*		85.4 - 91.5, 0.09<br>Belongs to *Nocardia cyriacigeorgica*
MDA3732 (NZ_PSZF00000000)	*94.32*	96.97	Not determined[6]	*0.82*		83.9 - 90.3, 0.03<br>Belongs to *Nocardia cyriacigeorgica*

[1]The reference breakpoints (for *gyr*B, ANI, AAI, and DDH-estimate and differences in G+C content) for assigning membership to a specific species are indicated in brackets. [2]Values lower than the reference breakpoints, suggestive of a distinct species, are indicated in italics. [3]Average nucleotide identity (ANI) determined using the TrueBac™ system (https://www.truebacid.com/genome/). [4]Average amino acid identity (AAI) determined by the AAI-profiler system

(http://ekhidna2.biocenter.helsinki.fi/AAI). [5]DDH-estimate and the difference in genomic GC content determined by the Type Strain Genome Server (TYGS) method (https://tygs.dsmz.de). [6]TrueBacTM, is a commercial web server (only the above-mentioned strains could be examined without cost).

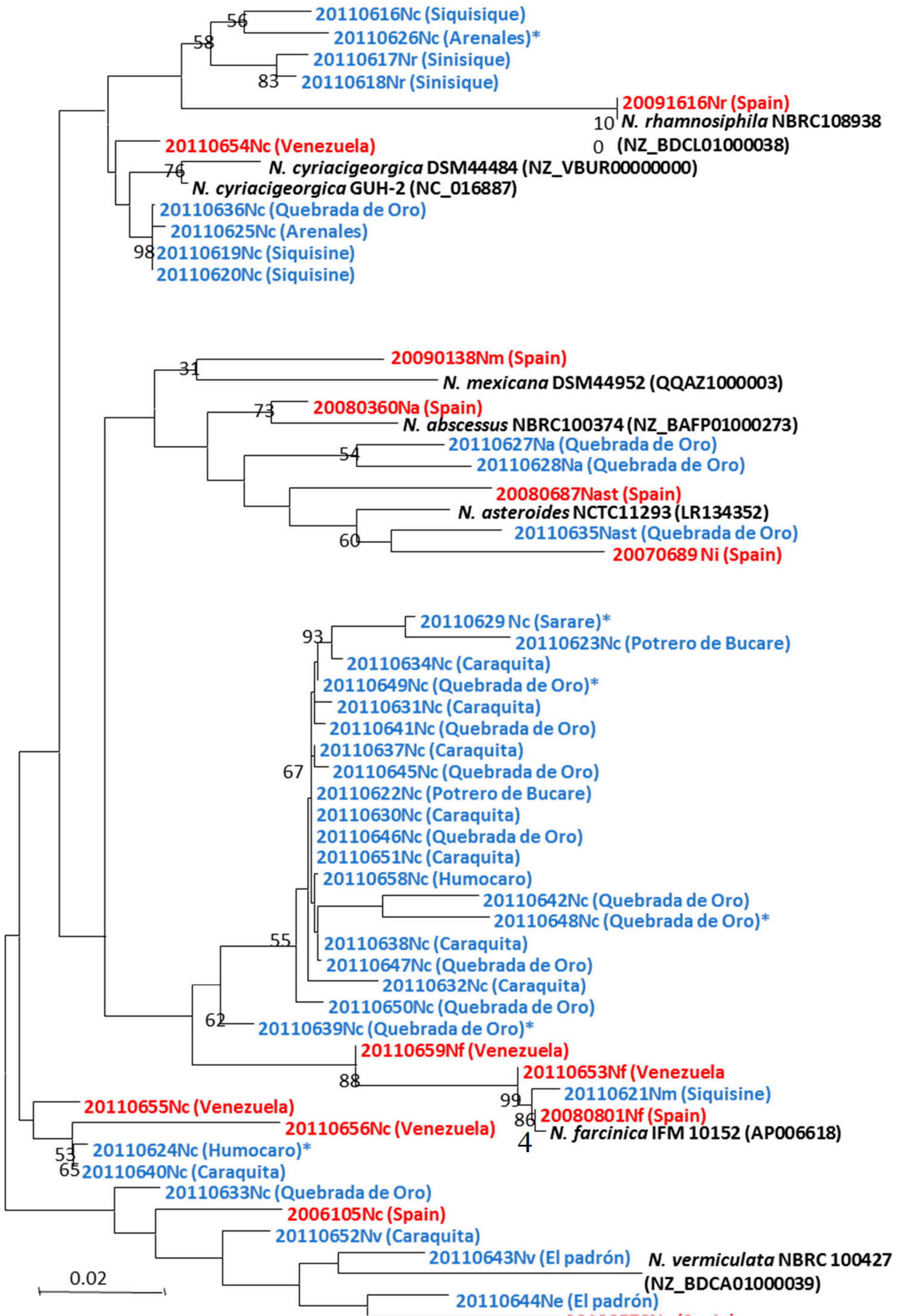
20110616Nc (Siquisique)
20110626Nc (Arenales)*
20110617Nr (Sinisique)
20110618Nr (Sinisique)
20091616Nr (Spain)
N. rhamnosiphila NBRC108938 (NZ_BDCL01000038)
20110654Nc (Venezuela)
N. cyriacigeorgica DSM44484 (NZ_VBUR00000000)
N. cyriacigeorgica GUH-2 (NC_016887)
20110636Nc (Quebrada de Oro)
20110625Nc (Arenales)
20110619Nc (Siquisine)
20110620Nc (Siquisine)
20090138Nm (Spain)
N. mexicana DSM44952 (QQAZ1000003)
20080360Na (Spain)
N. abscessus NBRC100374 (NZ_BAFP01000273)
20110627Na (Quebrada de Oro)
20110628Na (Quebrada de Oro)
20080687Nast (Spain)
N. asteroides NCTC11293 (LR134352)
20110635Nast (Quebrada de Oro)
20070689 Ni (Spain)
20110629 Nc (Sarare)*
20110623Nc (Potrero de Bucare)
20110634Nc (Caraquita)
20110649Nc (Quebrada de Oro)*
20110631Nc (Caraquita)
20110641Nc (Quebrada de Oro)
20110637Nc (Caraquita)
20110645Nc (Quebrada de Oro)
20110622Nc (Potrero de Bucare)
20110630Nc (Caraquita)
20110646Nc (Quebrada de Oro)
20110651Nc (Caraquita)
20110658Nc (Humocaro)
20110642Nc (Quebrada de Oro)
20110648Nc (Quebrada de Oro)*
20110638Nc (Caraquita)
20110647Nc (Quebrada de Oro)
20110632Nc (Caraquita)
20110650Nc (Quebrada de Oro)
20110639Nc (Quebrada de Oro)*
20110659Nf (Venezuela)
20110653Nf (Venezuela
20110621Nm (Siquisine)
20080801Nf (Spain)
N. farcinica IFM 10152 (AP006618)
20110655Nc (Venezuela)
20110656Nc (Venezuela)
20110624Nc (Humocaro)*
20110640Nc (Caraquita)
20110633Nc (Quebrada de Oro)
2006105Nc (Spain)
20110652Nv (Caraquita)
20110643Nv (El padrón)
N. vermiculata NBRC 100427 (NZ_BDCA01000039)
20110644Ne (El padrón)
0.02
56
58
83
100
76
98
31
73
54
60
93
67
55
62
88
99
86
53
65

Figure S1. Phylogenetic ML tree based on the MLSA analysis *gyr*B-16S rRNA-*sec*A-*hsp*65 genes) of the 38 *N. cyriacigeorgica* strains from soil (in blue), 5 *Nocardia* clinical strains from Venezuelan patients and nine Spanish clinical strains representing each species present in soil (in red), plus the type strains (in black). The asterisk indicates the strains selected for WGS. Na stands for *N. abscessus*, so on Nast for *N. asteroides*, Nc for *N. cyriacigeorgica*, Ne for *N. elegans*, Nf for *N. farcinica*, Ni for *N. ignorata*, Nm for *N. mexicana*, Nn for *N. nova*, Nr for *N. rhamnosiphila*, and Nv for *N. vermiculata*. The reliability of the topologies was assessed by the bootstrap method with 1000 replicates.

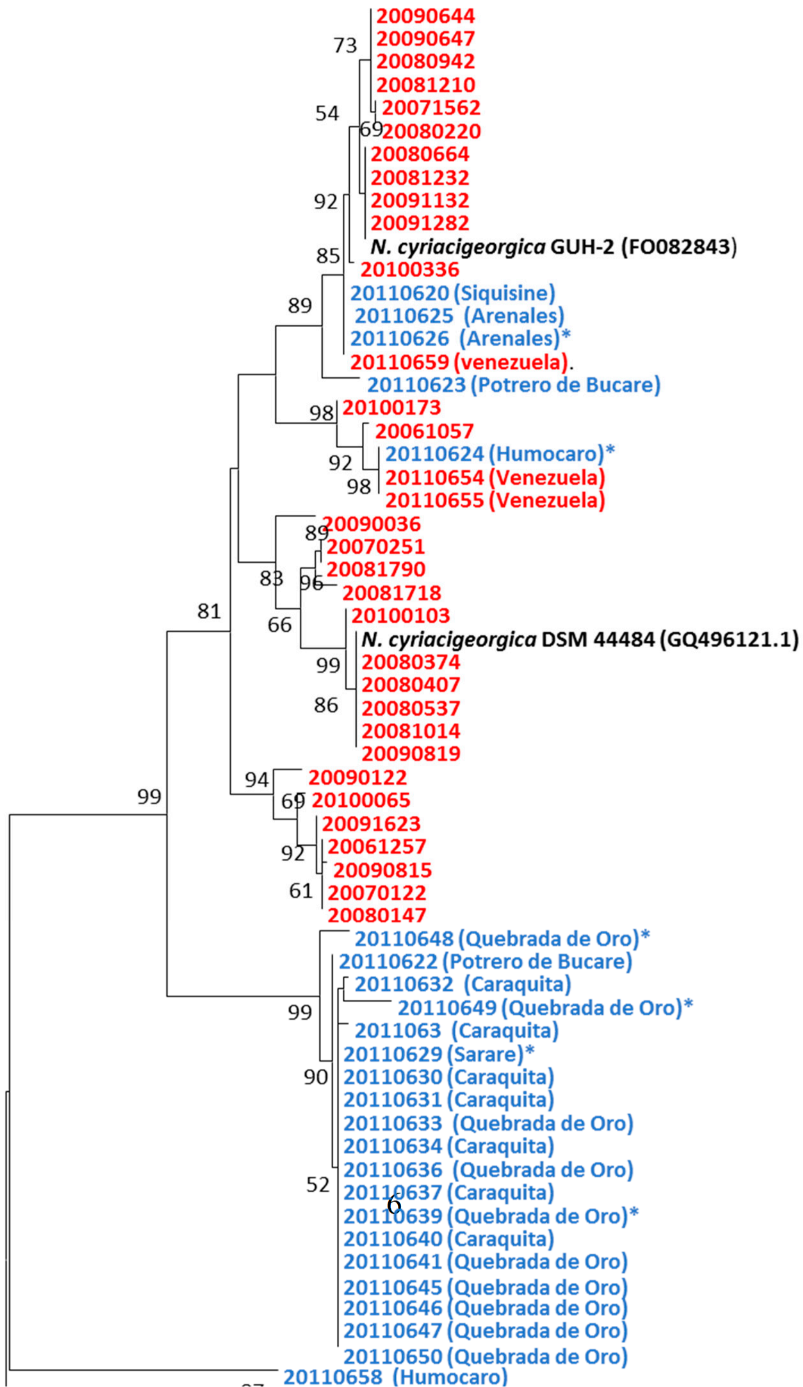
20090644
20090647
20080942
20081210
20071562
20080220
20080664
20081232
20091132
20091282
N. cyriacigeorgica GUH-2 (FO082843)
20100336
20110620 (Siquisine)
20110625 (Arenales)
20110626 (Arenales)*
20110659 (venezuela).
20110623 (Potrero de Bucare)
20100173
20061057
20110624 (Humocaro)*
20110654 (Venezuela)
20110655 (Venezuela)
20090036
20070251
20081790
20081718
20100103
N. cyriacigeorgica DSM 44484 (GQ496121.1)
20080374
20080407
20080537
20081014
20090819
20090122
20100065
20091623
20061257
20090815
20070122
20080147
20110648 (Quebrada de Oro)*
20110622 (Potrero de Bucare)
20110632 (Caraquita)
20110649 (Quebrada de Oro)*
2011063 (Caraquita)
20110629 (Sarare)*
20110630 (Caraquita)
20110631 (Caraquita)
20110633 (Quebrada de Oro)
20110634 (Caraquita)
20110636 (Quebrada de Oro)
20110637 (Caraquita)
20110639 (Quebrada de Oro)*
20110640 (Caraquita)
20110641 (Quebrada de Oro)
20110645 (Quebrada de Oro)
20110646 (Quebrada de Oro)
20110647 (Quebrada de Oro)
20110650 (Quebrada de Oro)
20110658 (Humocaro)
73
54
69
92
85
89
98
92
98
89
83
96
66
81
99
86
94
69
92
61
99
99
90
52

Figure S2. Phylogenetic relationships of the 29 Venezuelan *N. cyriacigeorgica* soil strains (in blue), three Venezuelan and 30 Spanish *N. cyriacigeorgica* clinical strains (in red), as revealed by their *gyr*B genes. The reliability of the ML topologies was assessed by the bootstrap method (1000 replications). The asterisk indicates the strains selected for WGS.

```
                  10        20        30        40        50        60        70        80        90       100
          ....|....|....|....|....|....|....|....|....|....|....|....|....|....|....|....|....|....|....|....|
GUH-2     MTETTLPPNGGAGDRIEPVDIQNEMQSSYIDYAMSVIVGRALPDVRDGLKPVHRRVLYAMYDNGYRPDRGYVKSARPVAETMGNYHPHGDASIYDTLVRM
DSM44484  ....................................................................................................
20110626  ....................................................................................................
20110624  ....................................................................................................
20110629  ..........................................................................................S.........
20110639  ..........................................................................................S.........
20110648  ..........................................................................................S.........
20110649  ..........................................................................................S.........

                 110       120       130       140       150       160       170       180       190       200
          ....|....|....|....|....|....|....|....|....|....|....|....|....|....|....|....|....|....|....|....|
GUH-2     AQPWSLRYPLVDGQGNFGSRGNDGAAAMRYTECRLTPLAMELLREIDHETVDFTPNYDGRSQEPVVLPSRVPNLLMNGSNGIAVGMATNIPPHNLNELAE
DSM44484  ....................................................................................................
20110626  ....................................................................................................
20110624  ...................................................................................................D
20110629  .........................................M...........V..........T......A............................
20110639  .........................................M...........V..........T......A............................
20110648  .........................................M...........V..........T......A............................
20110649  .........................................M...........V..........T......A............................

                 210       220       230       240       250       260       270       280       290       300
          ....|....|....|....|....|....|....|....|....|....|....|....|....|....|....|....|....|....|....|....|
GUH-2     AIYWALENYDADEEATLAACMERVKGPDFPTAGLIVGGQGIHDAYTTGRGSIRMRGVVEIEEDTRGRTTIVITELPYQVNTDNFINSIAEQVKDGKIAGI
DSM44484  ....................................................................................................
20110626  ....................................................................................................
20110624  ....................................................................................................
20110629  ......D..E....S................................................S....................................
20110639  ......D..E....S................................................S....................................
20110648  ......D..E....S................................................S....................................
20110649  ......D..E....S................................................S....................................

                 310       320       330       340       350       360       370       380       390       400
          ....|....|....|....|....|....|....|....|....|....|....|....|....|....|....|....|....|....|....|....|
GUH-2     SDIHDESSDRAGMRIVVTVKRDAVAKVVLNNLYKHTQLQTSFGANMLSIVDGVPRTLRLDQMIRYYVEHQLEVIVRRTRYLLRKAEERAHILRGLVKALD
```

```
DSM44484  ....................................................................................................
20110626  ....................................................................................................
20110624  ....................................................................................................
20110629  ................................................................F...................................
20110639  ................................................................F...................................
20110648  ................................................................F...................................
20110649  ................................................................F...................................

                 410       420       430       440       450       460       470       480       490       500
          ....|....|....|....|....|....|....|....|....|....|....|....|....|....|....|....|....|....|....|....|
GUH-2     ALDEVIALIRRSANTDTARTGLMQLLDIDEIQATAILDMQLRRLSALERQKIIDELAKIEAEIADLKDILDKPERQRAIVRDELAEIVEKYGDERRTKII
DSM44484  ....................................................V........................G......................
20110626  ....................................................................................................
20110624  ....................................................V...............................................
20110629  .......................D..E.........................V.................E.............................
20110639  .......................D..E.........................V.................E.............................
20110648  .......................D..E.........................V.................E.............................
20110649  .......................D..E.........................V.................E.............................

                 510       520       530       540       550       560       570       580       590       600
          ....|....|....|....|....|....|....|....|....|....|....|....|....|....|....|....|....|....|....|....|
GUH-2     AADGDVADEDLIAREDVVVTITETGYAKRTKTDLYRSQKRGGKGVQGAGLKQDDLVKHFFITSTHDWLLFFTNKGRVYRAKAYELPEANRTARGQHVANL
DSM44484  ....................................................................................................
20110626  ....................................................................................................
20110624  ....................................................................................................
20110629  .............................................................S..........................S...........
20110639  .............................................................S..........................S...........
20110648  .............................................................S..........................S...........
20110649  .............................................................S..........................S...........

                 610       620       630       640       650       660       670       680       690       700
          ....|....|....|....|....|....|....|....|....|....|....|....|....|....|....|....|....|....|....|....|
GUH-2     LAFQPDEKIAQIIRIKSYEDAPYLVLATKNGLVKKSRLSDFDSNRSGGIVAVNLRDNDELVGAVLCSADDDLLLVSAQGQSIRFAATDEALRPMGRATSG
DSM44484  ....................................................................................................
20110626  ....................................................................................................
20110624  ....................................................................................................
20110629  ....................................................................................S...............
20110639  ....................................................................................S...............
20110648  ....................................................................................S...............
20110649  ....................................................................................S...............
```

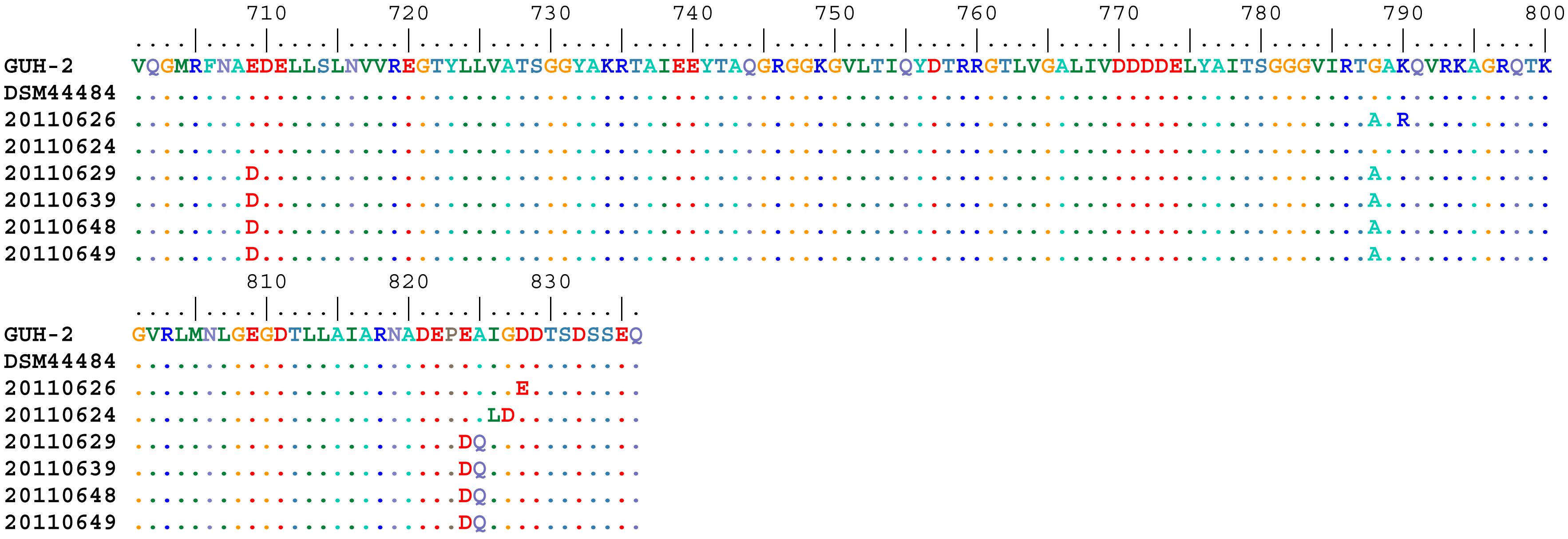

Figure S3. Amino acid sequences of GyrA alignment from *N. cyriacigeorgica* genome reference strain GUH-2, type strain DSM44484^T, CNM20110626, and CNM20110624 with major allele GyrA1, and the strains of the soil-only cluster (CNM20110629, CNM20110639, CNM20110648, and CNM20110649) with major allele GyrA2.

Material and methods. Soil *Nocardia spp.* culture and presumptive identification

The collection, culture, and presumptive identification were carried out by microbiologists of the Microbiology and Parasitology Department of the Faculty of Pharmacy and Bioanalysis (Los Andes University, Mérida-Venezuela).

This appendix includes the method previously published in Spanish by Ramírez A, Blanco M and García E in 2003 titled "Biogeografía de *Nocardia*: Estudio de la población edáfica de *Nocardia* en diversas zonas climáticas del Estado de Lara". Rev Soc Ven Microbiol. 23:1-7

Soil samples each weighing ≈300 g were collected randomly from a depth of 10 cm, after removing the surface. The samples were transferred aseptically to sterile bags and maintained at 4ºC after recollection. The loam and the sandy-clay loam were sifted through (2mm/10 mesh), handily homogenized for 15 minutes and dried at 105° C for 3 days. Each 50g of this sample was added to 450ml of charcoal-free broth [1] and gently shaken for 20 minutes. Later, the sediment of 10 minutes conforming to the 10^{-1} dilution was serial diluted to 10^{-8} [2].

From each 10^{-8} dilution, five aliquots of (1 ml each tube) were incubated at 34 ºC for five weeks including a paraffin dipstick and nine milliliters of CFB. As previously is described, the paraffin batting technique is based on the ability of the *Nocardia* genus to use paraffin as an only Carbon source [3]. The emergent colonies were grown in nutrient agar and identified as *Nocardia* genus by: phenotypic analysis of macro- and microscopic characteristics, Gram stain and degree of fragmentation of the hyphae (microculture), lysozyme resistance, presence of methyl esters of mycolic acids by thin-layer chromatography on silica gel [4]; and by the analysis of hydrolysis or decomposition of casein, xanthine, hypoxanthine, tyrosine and urea [5].

References

1. McClung N. Isolation of *Nocardia asteroides* from soils. *Mycology.* **1960**. *52*: 154-156.
2. Ramirez, A.; Blanco, M.; Garcia, E. Biogeografía de *Nocardia*: Estudio de la población edáfica de *Nocardia* en diversas zonas climáticas del estado Lara, Venezuela. *Rev Soc Ven Microbiol*. **2003**.*23*:142-147
3. Shawar, R.M; Moore, D.G.; LaRocco, M.T. Cultivation of *Nocardia* spp. on chemically defined media for selective recovery of isolates from clinical specimens. *J Clin Microbiol*. **1990**. *28*:508-512.
4. Hamid, M.E.; Minnikin, D.E.; Goodfellow, M.A. Simple chemical test to distinguish mycobacteria from other mycolic-acid-containing actinomycetes. *J Gen Microbiol*. **1993**. *139*:2203-2213.
5. Brown-Elliott BA, Brown JM, Conville PS, Wallace RJ. Clinical and laboratory features of the *Nocardia.* **2006**
